# Understanding the Motivations, Perceptions and Nutritional Implications of Plant‐Based Milk Consumption Compared to Dairy‐Based Milk

**DOI:** 10.1111/jhn.70254

**Published:** 2026-04-27

**Authors:** Isobel Harmer, Joel C. Craddock, Anita Lawrence, Tracy McCaffery, Katherine Kent, Karen E. Charlton

**Affiliations:** ^1^ School of Medical, Indigenous and Health Sciences, Faculty of Science, Medicine and Health University of Wollongong Wollongong New South Wales Australia; ^2^ School of Agriculture, Food and Ecosystem Sciences The University of Melbourne Parkville Victoria Australia; ^3^ Department of Nutrition, Dietetics and Food Monash University Melbourne Victoria Australia; ^4^ School of Health Sciences University of Newcastle Callaghan New South Wales Australia; ^5^ Nutrition and Metabolic Health Research Program Hunter Medical Research Institute New Lambton New South Wales Australia

**Keywords:** cow's milk, nutritional adequacy, perceptions, plant‐based milk, vegetarian‐style eating

## Abstract

**Background:**

Plant‐based milks have been increasing in popularity amongst Australian consumers, concurrent with a decrease in cow's milk consumption. Given the key role of cow's milk in Australian diets, it is necessary to understand the motivations behind this consumer behaviour and investigate the nutritional implications associated with this shift in dietary choice.

**Methodology:**

Adults, including both omnivores and purposefully targeted vegan individuals, were recruited via social media to complete an online survey and two 24‐h dietary recalls using the online Intake24 dietary assessment programme. The survey explored milk type choice and participant perceptions of the health and environmental impact of plant‐based milks. Respondents were divided into groups based on whether they reported to consume or not consume dairy products in the survey. Survey and dietary intake data were then compared between these two groups.

**Results:**

Of the 217 survey responses received (*n* = 74 dairy and *n* = 143 non‐dairy consumers), soy, almond and oat plant‐based milks were the most popular choices. The primary drivers behind milk type choice were animal rights, self‐reported adverse health symptoms and environmental concerns. Additionally, non‐dairy consumers were more likely to perceive these products as healthier and better for the environment than cow's milk. Dietary intake data identified that overall non‐dairy consumers had significantly lower intakes of saturated fat, iodine and vitamin B12 (14.9 vs. 21.9 g/day; *p* = 0.001, 70.8 vs. 128.8 μg/day; *p* < 0.001 and 0.9 vs. 3.0 μg/day; *p* < 0.001, respectively) and higher amounts of dietary fibre (27.2 vs. 21.3 g/day; *p* = 0.008) compared to dairy consumers.

**Conclusion:**

This study provides novel insights into the motivations to consume, and perceptions of the healthfulness, plant‐based milk in Australia and identified that non‐dairy consumers may be at increased risk of iodine and vitamin B12 deficiency.

## Summary

- Dietary intake data identified that those that do not consume dairy had significantly lower intakes of saturated fat, iodine and vitamin B12, and significantly higher intakes of dietary fibre, compared to dairy consumers.
- The most popular milk type choices of survey participants were soy, almond and oat plant‐based milks followed by skim cow's milk.
- The primary motivators behind milk type choice were identified as animal rights, self‐reported adverse health symptoms and environmental concerns.
- Non‐dairy consumers were more likely to perceive plant‐based milk products as healthier and better for the environment than cow's milk when compared to dairy consumers.

## 1 Introduction

Plant‐based milks are increasing in popularity globally and within Australia. The Australian Bureau of Statistics estimate a 30% increase in plant‐based milk consumption between 2019 and 2021 [1]. The reasons behind this shift in consumer demand remain largely unknown in the Australian context. Intolerance and allergies to dairy may be partly responsible for this consumer change, given their high global prevalence [2, 3], however, reasons for choosing plant‐based milk are likely more complex. Yantcheva and colleagues reported that the primary reason why many Australian adults were choosing to avoid dairy foods [4] was self‐reported digestive symptoms (e.g., bloating or abdominal pain), generally in the absence of a medical diagnosis such as allergy or intolerance. Other reasons included cardiovascular disease diagnosis, personal health reasons (e.g., goal of weight loss) or a dislike of cow's milk taste. Notably, this research did not explore whether individuals were drinking plant‐based milk in place of dairy cow's milk, nor investigate consumer opinions on plant‐based milk and whether choices were linked to environmental and planetary health concerns [4].

There has been some international investigation into the perceptions and motivations behind, specifically, plant‐based milk purchasing. A study by Martinez‐Padilla and colleagues in Denmark surveyed young adults (16–35 years) about their perceptions of plant‐based milks and found that plant‐based milk consumers were more likely than non‐consumers to perceive plant‐based milks as good for health, nutritionally equivalent or nutritionally better than cow's milk, and as environmentally friendly products [5]. Similarly, a study by Su and colleagues in China identified that individuals that were concerned about their own health and the state of the environment were more likely to perceive plant‐based milk as superior to cow's milk for both of these metrics, and would therefore be willing to pay more for these products [6]. Two US‐based studies found a relationship between choice for plant‐based milk over dairy, and individual values for personal health, animal welfare and environmental concerns [7, 8].

However, the methodology of the two US studies provides considerable opportunity for bias with one study by McCarthy et al. [7] having a goal of increasing dairy milk sales, and the other by Boaitey and Minegishi [8] recruiting participants attending an agricultural fair. Furthermore, many of these international studies included primarily female and highly educated participants which do not reflect the demographics of their general populations. Although conducted overseas and with potential biases in population sampling, these investigations highlight factors that may also be contributing to Australians' reasons for choosing, and their perceptions of, plant‐based milk.

The current research into perceptions of consumers that choose plant‐based milk over dairy identify health as a recurring theme, and it is important to consider this in the context of major differences in the nutritional composition of plant‐based milk. Studies conducted outside of Australia have identified, that compared to cow's milk, unfortified plant‐based milk is typically lower in saturated fat, energy, calcium, zinc, iodine, phosphorus and vitamins A, D, B_2_ and B_12_ [9, 10, 11, 12, 13, 14]. Protein content is also lower in all unfortified plant‐based milk types except for legume‐based soy and pea beverages [9, 10, 11, 12, 13, 14, 15]. Although limited studies have explored plant‐based milk micronutrient content in extensive detail, one Swiss paper by Walther and colleagues identified that soy milk contained more of the vitamins B_1_, B_6_, E, K and folic acid, as well as more magnesium, manganese, iron and copper when compared to other plant‐based milks suggesting it to be the most micronutrient dense as well as protein rich of the plant based milks [16]. Australian research comparing the nutrient composition between cow's milk and plant‐based milks has identified that, in general, the plant‐based milks available are significantly lower in protein, total fat, saturated fat, sugar, iodine, zinc and vitamins A, B_2_ and B_12_ when compared to cow's milk [17, 18]. As cow's milk provides more than 5% of total daily nutrient intake in Australia [19] direct replacement of cow's milk with plant‐based milk may alter the balance of nutrients in the diet.

Another nutrient identified to be lower in Australian plant‐based milks is iodine [17, 18], a micronutrient that is at risk of suboptimal intake in some Australian populations, including women of childbearing age [20, 21, 22, 23]. Mandatory bread fortification with iodised salt was introduced in 2009 [24], based on an estimated consumption of 100 g of bread/day [25]. However, a secondary analysis of the National Nutrition and Physical Activity 2011–12 Survey (NNPAS) reported that only 8.6% of Australian women of child‐bearing age reported consuming ≥ 100 g of bread/day [26, 27], highlighting the important contribution of dairy products, primarily cow's milk, as remaining the major source of iodine in the Australian diet. It is important to understand whether the differences in nutrient composition of plant‐based milk and cow's milk translate to total nutrient intake differences when the diet is considered as a whole. Australian research that *modelled* the substitution of cow's milk with plant‐based milk found a decreased intake of calcium, iodine, phosphorus, zinc and vitamins B_2_ and B_12_ [19]. However, to the author's knowledge, there is no recent Australian research comparing *actual* reported dietary nutrient intake of plant‐based milk and dairy milk consumers. That is, whilst dietary modelling shows a decreased intake of several nutrients when plant‐based milk replaces dairy milk, non‐dairy consumers may select other foods or supplements that compensate for nutritional deficits. Given the role of cow's milk in Australian diets, this study aims to investigate the motivations behind milk type choice, whilst also gaining an understanding of the differences in micronutrient intake between dairy and plant‐based milk (non‐dairy) consumers according to actual dietary consumption patterns.

## 2 Methods

### 2.1 Study Overview

The cross‐sectional observational study comprised two parts, an online survey related to consumer perceptions, followed by an online 24‐h recall completed on two separate occasions.

Participants were adults aged 18 y and over that spoke English and resided in Australia. A participant information sheet and a survey link were posted on social media platforms, including Facebook and LinkedIn. Those following vegetarian and vegan dietary patterns were purposefully recruited through networks such as vegan community pages as they were considered to be a sector of the population highly likely to be plant‐based milk consumers. Snowball recruitment was utilised where, after completing the survey, respondents were encouraged to share the survey with their peers, regardless of milk type preference [28]. Ethics approval was gained through the University of Wollongong Human Research Ethics Committee (HREC 2023/044).

### 2.2 Online Survey

The survey instrument underwent construct and face validity using a convenience sample of four academics external to the project with experience in survey development. The validation framework was based on evidence‐informed approaches for survey validation [29, 30, 31]. The survey (see Supporting Material S1) was hosted on the platform REDCap [32] and comprised 20 questions, including multiple‐choice and short‐response questions. It was separated into five sections: (1) introduction and PIS; (2) participant demographics and dietary pattern information; (3) milk consumption questions including specific plant‐based milk brand and product types; (4) motivations behind milk choice consumption and perceptions about plant‐based milk health and environmental impact; (5) link to Intake24‐Australia (details below), an online 24‐h recall on the day of survey and 8 days afterwards. Incomplete survey responses were identified as those where no questions of survey sections three (milk choice) and four (motivations and perceptions) were completed. All valid survey responses were used when examining survey data despite drop out continuing to the dietary recall. The Checklist for Reporting Results of Internet E‐surveys was applied during survey development to ensure that result reporting is transparent [33].

### 2.3 Intake24

Dietary intake was assessed using Intake24, an online self‐completed 24‐h recall, that followed directly from the survey. Intake24 was originally developed in the United Kingdom [34, 35] and has since been adapted for the Australian population and validated in a feeding study [36, 37, 38]. It has also been validated using absolute and relative methods in English‐speaking participants aged 11–88 y [39, 40, 41].

Participants were asked to complete two 24‐h recalls to account for day‐to‐day variation [42]. A direct link to the first recall on Intake24, along with detailed instructions on completing it and specific details to describe milk intake was provided. Eight days later, REDCap automatically sent participants the link to their second recall [32].

Intake24 utilises the multiple pass method for 24‐h recall instrument and is linked to the AUSNUT 2011–2013 food composition database [43]. No information on supplement use was collected. Items identified as missing foods when participants were entering their recall were reviewed by a research team member and matched with an existing food from the AUSNUT 2011–2013 database [43]. All matched missing foods and substitutions were reviewed by an accredited practising dietitian. For foods that could not be matched, the closest recipe from the ‘AUSNUT 2011–2013 food recipes file’ [44] was utilised as a base and relevant substitutions were made. For reported plant‐based versions of traditional animal product‐containing foods, the animal product portions were replaced with an equal amount of a plant‐based alternative by the research team. Nutrient values provided for plant‐based milk and other dairy alternatives are limited in the database and do not accurately reflect the variety and nutritional composition of products available in today's Australian market [45]. The authors conducted a supermarket audit in 2023 [18] and this up‐to‐date nutrition composition information was incorporated into the current dietary survey data analysis. This included plant‐based milk and dairy alternatives found in coffees, smoothies or other recipes such as porridge. For plant‐based milk and dairy alternatives reported in mixed commodity foods, the ‘AUSNUT 2011–13 food recipes file’ [44] recipe quantities were used to substitute plant‐based milk.

An average daily nutrient consumption was calculated for participants (*n* = 97) who completed two valid recalls. For participants who completed only one valid 24‐h recall, the singular recall was used to assess daily nutrient intake. Herein, for all participants, dietary intakes are referred to as average intakes, average daily intakes or median daily intakes, as appropriate.

### 2.4 Data Cleaning and Analysis

Data were reviewed and cleaned within Microsoft Excel (version 16.77) following standard monitoring checks (see Resources section at https://intake24.com/resources), with invalid recalls removed from the analysis [46]. Reasons for removal included recalls with < 10 items, or those with unrealistic average total daily energy intake (< 40 kcal/d or > 4000 kcal/d). Statistical analysis was performed using IBM SPSS Statistics for Windows software (Version 26.0, 2017 Armonk, NY: IBM Corp.). Valid data from REDCap and Intake24‐Australia were matched to participants by participant number and combined for analysis. Shapiro–Wilk tests were used to assess data normality. Comparisons were made between dairy and non‐dairy consumers, as well as to women of childbearing age (18–45 y). Mann–Whitney *U* tests and exact two‐tailed *p* value were performed to compare nutrition consumption data between groups for non‐parametric data and were presented as a median and interquartile range. Demographic variables were compared between dairy and non‐dairy consumers using Pearson's *χ* ^2^ test. Significance was set at *p* < 0.05.

## 3 Results

**Figure 1:**
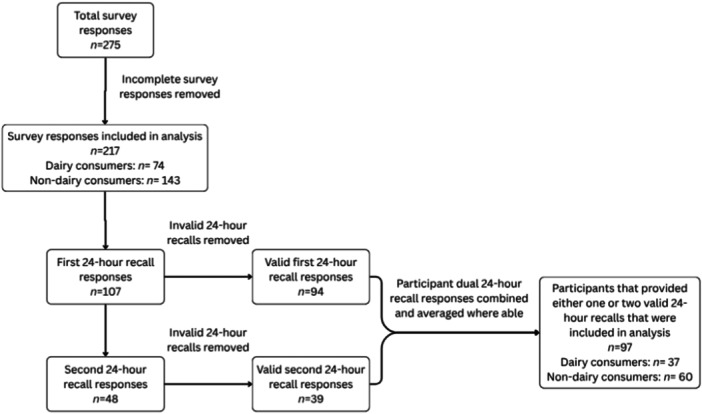
Flowchart representing the response rate for the survey and Intake24.

In total, there were 275 responses to the survey, with 217 of these deemed to be eligible for analysis. Of the 217 survey respondents, 94 individuals completed a valid first 24‐h recall and 39 individuals completed a valid second 24‐h recall, after combining and averaging dual participant responses where able, there were 97 completed records for either 1 or 2 days (Figure 1). Of the dairy consumers that completed valid 24‐h recalls, 38.5% completed two valid 24‐h recalls and 61.5% completed only one. Among the non‐dairy consumers that completed valid 24‐h recalls, 25.5% completed two valid recalls and 74.5% completed one.

### 3.1 Demographic Characteristics

Demographic characteristics of participants are shown in Table 1. Participants were primarily female (79.3%) with a median age of 34 y [27, 28, 29, 30, 31, 32, 33, 34, 35, 36, 37, 38, 39, 40, 41]. Most respondents lived in the state of New South Wales (52.2%) and were Australian or Caucasian background (*n* = 112 and 85, respectively). Most participants had completed university‐level education (38.2% at Bachelor's degree level; 30.4% with masters or honours) and median body mass index was 24.0 kg/m^2^ (21.7–26.4 kg/m^2^). There was a significant association between education level and milk group (*χ* ^2^(5) = 16.79, *p* < 0.01) with dairy consumers more commonly holding the highest education qualification, and plant‐based milk drinkers more commonly represented in lower education categories. Comparisons were not conducted for the ‘animal product‐based food groups avoided’ variable, as vegan and vegetarian participants were purposefully targeted for recruitment, and avoidance of various animal‐based products, including cow's milk, was therefore expected within this cohort.

**Table 1:** Demographic characteristics of survey participants (*n* = 217).

		Descriptive statistic			
Characteristic	Category	Dairy consumer (*n* = 74)	Non‐dairy consumers (*n* = 143)	Total (*n* = 217)	*p*
Age in years	—	34 (28–39)	35 (27–42)	34 (27–41)	0.56
median (Q1–Q3)					
Gender *n* (%)	Male	11 (14.9%)	28 (19.6%)	39(18.0%)	0.77
	Female	61 (82.4%)	111 (77.6%)	172 (79.3%)	
	Non‐binary	1 (1.35%)	3 (2.1%)	4 (1.8%)	
	Prefer not to say	1 (1.35%)	1 (0.7%)	2 (0.9%)	
Body mass index (kg/m^2^)	—	24.5 (22.2–27.1)	23.7 (21.5–26.0)	24.0 (21.7–26.4)	0.10
median (Q1–Q3)^a^					
Location^b^ *n* (%)	New South Wales	55 (76.3%)	91 (64.0%)	145 (52.2%)	0.10
	Victoria	3 (4.2%)	14 (9.9%)	17 (6.1%)	
	Queensland	3 (4.2%)	13 (9.2%)	16 (5.8%)	
	Australian Capital Territory	3 (4.2%)	12 (8.5%)	16 (5.8%)	
	Tasmania	4 (5.6%)	3 (2.1%)	7 (2.5%)	
	Western Australia	0 (0.0%)	6 (4.2%)	6 (2.2%)	
	South Australia	3 (4.2%)	2 (1.4%)	5 (1.8%)	
	Northern Territory	1 (1.4%)	1 (0.7%)	2 (0.7%)	
Race/ethnicity *n* (%)	Caucasian	35 (16.1%)	74 (34%)	109 (50.2%)	0.44
	Australian	37 (17.1%)	52 (24.0%)	89 (41.0%)	
	Asian	3 (1.4%)	8 (3.7%)	11 (5.1%)	
	European	2 (0.9%)	14 (6.5%)	16 (7.4%)	
	Oceania (outside Australia)	2 (0.9%)	1 (0.5%)	3 (1.4%)	
	North America	0 (0.0%)	2 (0.9%)	2 (0.9%)	
	South America	1 (0.5%)	0 (0.0%)	1 (0.5%)	
Highest level of education (completed or enroled) *n* (%)	High school	3 (4.1%)	14 (9.8%)	17 (7.8%)	< 0.01
	Post‐school diploma or equivalent	6 (8.1%)	18 (12.6%)	24 (11.1%)	
	University bachelor's/undergraduate degree	25 (33.8%)	58 (40.6%)	83 (38.2%)	
	University honours/masters degree	22 (29.7%)	44 (30.8%)	66 (30.4%)	
	University (doctorate degree)	17 (23.0%)	9 (6.3%)	26 (12%)	
	Prefer not to say	1 (1.4%)	0 (0.0%)	1 (0.5%)	
Animal product‐based food groups that participants do not consume	Red meat	22 (29.7%)	138 (96.5%)	160 (73.7%)	—
	Poultry (chicken, turkey, duck, etc.)	19 (25.7%)	137 (95.8%)	156 (71.9%)	
	Fish/seafood	17 (23.0%)	136 (95.1%)	153 (70.5%)	
	Eggs	3 (4.1%)	132 (92.3%)	135 (62.2%)	
	Honey	5 (6.8%)	106 (74.1%)	111 (51.1%)	
	I eat all of the above	45 (60.8%)	0 (0.0%)	45 (20.7%)	

^a^ BMI (kg/m^2^) calculated from participant‐reported weight (kg) and height (cm).

^b^ Based on participant‐reported post code.

### 3.2 Animal Product Consumption

When asked to report which animal product‐based food groups were not consumed, 65.9% of all respondents reported avoidance of dairy products. Of all survey respondents, only 20.7% consumed all the animal product‐based food groups listed. The majority of non‐dairy consumers (*n* = 143) also did not consume other animal product‐based food groups (between 74.1% and 96.5% of non‐dairy consumers) (Table 1).

### 3.3 Participant Milk Choice

Self‐reported milk choices of respondents are summarised in Table 2. Most (83.4%) participants consumed a plant‐based milk as their primary milk choice, with only 16.1% of respondents indicating that cow's milk was their primary choice. This included 47.3% of dairy consumers identifying a plant‐based milk as their primary milk of choice. The most popular milk type choice overall was soy milk (consumed by 74.5%), with 42.6% of participants reporting it as their primary milk of choice. Next was oat and almond plant‐based milks (25.9% and 13.0% of participants, respectively, reporting these as their primary milk choice), and lastly cow's milk (7.9% reporting low‐fat cow's milk and 6.0% reporting full‐fat cow's milk).

**Table 2:** Milk types consumed by survey participants.

	Participants that selected *n* (%)					
	All milk choices^a^			Primary milk choice^b^		
Milk type	Dairy consumers	Non‐dairy consumers	Total	Dairy consumers	Non‐dairy consumers	Total
	(*n* = 74)	(*n* = 143)	(*n* = 217)	(*n* = 74)	(*n* = 143)	(*n* = 217)
Low‐fat cow's milk	34 (45.9%)	0 (0.0%)	35 (16.1%)	17 (23.0%)	0 (0.0%)	17 (7.8%)
Full‐fat cow's milk	36 (48.4%)	0 (0.0%)	36 (16.6%)	13 (17.6%)	0 (0.0%)	13 (6.0%)
Lactose‐free cow's milk	10 (13.5%)	0 (0.0%)	10 (4.6%)	3 (4.1%)	0 (0.0%)	3 (1.4%)
Skim cow's milk	22 (29.7%)	0 (0.0%)	23 (10.6%)	2 (2.7%)	0 (0.0%)	2 (0.9%)
Total cow's milk	—	—	—	35 (47.3%)	0 (0.0%)	35 (16.1%)
Soy milk	34 (45.9%)	127 (88.8%)	161 (74.2%)	13 (17.6%)	79 (55.2%)	92 (42.4%)
Oat milk	33 (44.6%)	115 (80.4%)	148 (68.2%)	13 (17.6%)	43 (30.1%)	56 (25.8%)
Almond milk	33 (44.6%)	86 (60.1%)	119 (54.8%)	11 (14.9%)	17 (11.9%)	28 (12.9%)
Rice milk	1 (1.4%)	15 (10.5%)	16 (7.4%)	0 (0.0%)	2 (1.4%)	2 (0.9%)
Cashew milk	0 (0.0%)	16 (11.2%)	16 (7.4%)	0 (0.0%)	1 (0.7%)	1 (0.5%)
Coconut milk	13 (17.6%)	54 (37.8%)	67 (30.8%)	0 (0.0%)	1 (0.7%)	1 (0.5%)
Combination milk (plant‐based)	3 (4.1%)	5 (3.5%)	8 (3.7%)	1 (1.4%)	0 (0.0%)	1 (0.5%)
Hemp milk	2 (2.7%)	6 (4.2%)	8 (3.7%)	0 (0.0%)	0 (0.0%)	0 (0.0%)
Macadamia milk	1 (1.4%)	18 (12.6%)	19 (8.8%)	0 (0.0%)	0 (0.0%)	0 (0.0%)
Pea milk	1 (1.4%)	5 (3.5%)	6 (2.8%)	0 (0.0%)	0 (0.0%)	0 (0.0%)
Total plant‐based milk	—	—	—	38 (51.3%)	143 (100%)	181 (83.4%)
Other	2 (2.7%)	1 (0.7%)	3 (1.4%)	0 (0.0%)	0 (0.0%)	0 (0.0%)
Non‐milk drinkers	0 (0.0%)	0 (0.0%)	0 (0.0%)	0 (0.0%)	0 (0.0%)	0 (0.0%)
No response provided	0 (0.0%)	0 (0.0%)	1 (0.5%)	1 (1.4%)	0 (0.0%)	1 (0.5%)

^a^ Multiple‐choice question where participants can select unlimited milk types.

^b^ Multiple choice question where participants can only select a single milk type.

When examining the fortification status of the specific plant‐based milks reported by 24‐h recall respondents (*n* = 97), 3.8% of plant‐based milks were unfortified, 26.3% were fortified with only calcium, and 23.8% were fortified with calcium and at least one other nutrient. For 46.3% of the reported plant‐based milks, participants provided insufficient information on the brand to determine fortification status.

### 3.4 Motivations Behind Milk Choice

Animal rights (63.6%), health (58.1%) and environmental concerns (49.8%) were the three most commonly reported reasons for choice of milk type (Table 3). When asked to report their primary reason for choice of milk, 47% reported animal rights, followed by health (13.4%) and intolerance/allergy/negative symptoms (e.g., bloating, diarrhoea, pain) to dairy milk that had not been diagnosed by a health practitioner (8.8%). Only 5.1% of respondents indicated that environmental concerns were the primary reason for their milk choice. The ‘other’ responses across both dairy and non‐dairy consumers were primarily related to taste preference or elaboration on one of the listed options.

**Table 3:** Participants' reasons for choice of milk type listed in order of primary reason selected (*n* = 217).

Potential reasons	Participants that selected *n* (%)					
	All reasons^a^			Primary reason^b^		
	Dairy consumers (*n* = 74)	Non‐dairy consumers (*n* = 143)	Total (*n* = 217)	Dairy consumers (*n* = 74)	Non‐dairy consumers (*n* = 143)	Total (*n* = 217)
Animal rights	18 (24.3%)	120 (83.9%)	138 (63.6%)	7 (9.5%)	95 (66.4%)	102 (47.2%)
Health	43 (58.1%)	83 (58.0%)	126 (58.1%)	14 (18.9%)	15 (10.5%)	29 (13.4%)
Due to intolerance/allergy/negative symptoms (e.g., bloating, diarrhoea, pain) to dairy milk that has NOT been diagnosed by a health practitioner	19 (25.7%)	19 (13.2%)	38 (17.5%)	13 (17.6%)	6 (4.2%)	19 (8.8%)
Other	11 (14.86%)	10 (7.0%)	21 (9.7%)	10 (13.5%)	3 (2.1%)	13 (6.0%)
I have always had the same type of milk	22 (29.7%)	4 (2.8%)	26 (12.0%)	10 (13.5%)	2 (1.4%)	12 (5.5%)
Environmental concerns	16 (21.6%)	92 (64.3%)	108 (49.8%)	4 (5.4%)	7 (4.9%)	11 (5.1%)
Due to intolerance/allergy/negative symptoms (e.g., bloating, diarrhoea, pain) to dairy milk that has been diagnosed by a health practitioner	4 (5.4%)	17 (11.9%)	21 (9.7%)	2 (2.7%)	7 (4.9%)	9 (4.1%)
Price	14 (18.9%)	19 (13.3%)	33 (15.2%)	4 (5.4%)	0 (0.0%)	4 (1.8%)
My friends and/or family do and it's easy for me	10 (13.5%)	3 (2.1%)	13 (6.0%)	3 (4.1%)	0 (0.0%)	3 (1.4%)
Weight loss	3 (4.1%)	9 (6.3%)	12 (5.5%)	0 (0.0%)	1 (0.7%)	1 (0.5%)
To improve my athletic performance	4 (5.4%)	7 (4.9%)	11 (5.1%)	0 (0.0%)	0 (0.0%)	0 (0.0%)
My religion or spiritual beliefs	0 (0.0%)	4 (2.8%)	4 (1.8%)	0 (0.0%)	0 (0.0%)	0 (0.0%)
It's trendy	1 (1.4%)	2 (1.4%)	3 (1.4%)	0 (0.0%)	0 (0.0%)	0 (0.0%)
My friends and/or family do so I feel pressure to as well	1 (1.4%)	0 (0.0%)	1 (0.5%)	0 (0.0%)	0 (0.0%)	0 (0.0%)
Prefer not to say	0 (0.0%)	0 (0.0%)	0 (0.0%)	0 (0.0%)	0 (0.0%)	0 (0.0%)
No response provided	0 (0.0%)	0 (0.0%)	14 (6.5%)	7 (9.5%)	7 (4.9%)	14 (6.5%)

^a^ A multiple‐choice question where participants can select unlimited responses.

^b^ A multiple‐choice question where participants can select only a single response.

### 3.5 Perceptions Regarding Milk Types

Non‐dairy consumers primarily perceived that plant‐based milks were healthier than cow's milk (77.4%), compared to only 14.7% of dairy consumers who expressed this opinion. A quarter of dairy consumers either reported that plant‐based milks were equally as healthy as dairy milks (25.0%), were unsure (23.5%), or else perceived dairy milk to be healthier than plant‐based milk (20.6%). The ‘other’ responses for both dairy and non‐dairy consumers were mainly that it depended on the specific type of plant‐based milk or cow's milk and depended on the definition of health being used. When respondents were asked ‘What do you think the health and nutrition differences, if any, are between plant milks and dairy milks’ a few key themes emerged (Supporting Material S2). In particular, both dairy and non‐dairy consumers identified nutrient differences between milk types and a need for fortification of plant‐based milks with some nutrients, while non‐dairy consumers expressed beliefs about negative health attributes of dairy milk and concern for the highly processed nature of some plant‐based milks.

Non‐dairy consumers generally (89%) believed that plant‐based milks were better for the environment than dairy milks. The responses from dairy consumers differed where 43.9% perceived plant‐based milk to be better than cow's milk, almost a quarter (21.2%) believed different milk types to have an equal environmental impact, while 15.2% responded ‘other’. ‘Other’ responses for both dairy and non‐dairy consumers were primarily that degree of environmental impact is dependent on the type of plant‐based milk, and that there are environmental issues with all milk types. When expanding on their beliefs in the open‐ended response question (Supporting Material S2), overall, respondents almost always perceived that plant‐based milks had a reduced impact on the environment, compared to cow's milk. No obvious differences emerged in common themes identified from dairy and non‐dairy consuming participants.

### 3.6 Nutrient Intake Analysis

Non‐dairy consumers had a significantly lower median daily intake than dairy consumers for the following nutrients: saturated fat (median 14.9 (Q1–Q3: 9.3–21.2) vs. 21.9 (14.7–28.0) g/day; *p* = 0.001); iodine (median 70.8 (50.4–106.8) vs. 128.8 (84.7–162.8) µg/day; *p* < 0.001); and vitamin B12 (median 0.9 (0.6–2.4) vs. 3.0 (1.8–3.8) µg/day; *p* < 0.001). Median dietary fibre intake was significantly higher in non‐dairy consumers (median 27.7 (20.5–34.7) vs. 21.3 (16.1–34.0) g/day; *p* = 0.008) (Table 4).

**Table 4:** Median (Q1–Q3) daily nutrient intake of dairy (*n* = 37) and non‐dairy consumers (*n* = 60).

Nutrient	Dairy consumers (*n* = 37)	Non‐dairy consumers (*n* = 60)	*p* a
Energy (kJ)	6571 (6046–8499)	6634 (4661–8094)	*p* = 0.334
Protein (g)	71.2 (51.3–89.7)	56.2 (44.5–84.3)	*p* = 0.069
Fat (g)	60.3 (51.4–84.3)	57.1 (40.4–76.3)	*p* = 0.334
Saturated fat (g)	21.9 (14.7–28.0)	14.9 (9.3–21.2)	*p* = 0.001^a^
Carbohydrate (g)	170.7 (147.0–209.2)	166.0 (120.8–217.8)	*p* = 0.435
Total sugars (g)	63.2 (51.3–86.1)	55.2 (34.6–80.5)	*p* = 0.188
Fibre (g)	21.3 (16.1–34.0)	27.7 (20.5–34.7)	*p* = 0.008^a^
Sodium (mg)	1616 (1474–2421)	1787 (1377–2547)	*p* = 0.731
Calcium (mg)	733 (609–1033)	692 (463–1009)	*p* = 0.938
Iodine (µg)	129 (85–163)	71 (50–107)	*p* < 0.001^a^
Zinc (mg)	8.5 (6.7–10.5)	7.4 (5.3–10.2)	*p* = 0.234
Phosphorus (mg)	1156 (958–1456)	991 (751–1389)	*p* = 0.229
Vitamin A retinol equivalents (µg)	577 (416–810)	511 (270–896)	*p* = 0.562
Riboflavin (Vitamin B2) (mg)	1.4 (1.0–1.6)	1.1 (0.7–1.7)	*p* = 0.073
Vitamin B12 (µg)	3.0 (1.8–3.8)	0.9 (0.6–2.4)	*p* < 0.001^a^

a A statistically significant (*p* < 0.05) difference between dairy and non‐dairy consumers using a Mann–Whitney *U* test means comparison with an exact two‐tailed *p* value.

Women of childbearing age (*n* = 67) had a similar daily iodine intake compared to all other participants (*n* = 30) (median 95 (67–135) µg/day vs. 86 (54–118) µg/day, respectively, *p* = 0.2251). There was also no difference between the daily iodine intake of women of childbearing age that did (*n* = 44) (102 (75–159) µg/day) and did not (*n* = 23) (93 (63–130) µg/day) consume dairy (*p* = 0.309).

## 4 Discussion

This online cross‐sectional observational survey investigated the milk choice, drivers for milk choice, and perceptions of plant‐based milk of 217 respondents. The respondents were primarily female, of a healthy weight and highly educated, but not representative of the general Australian population. The respondent demographics only differed between dairy and non‐dairy groups for education level, allowing for more effective comparison. Many of the respondents indicated that they do not consume at least one group of animal products (e.g., red meats, seafood and fish or dairy products), with 66% indicating they do not consume dairy products. The most popular primary milk type choices were soy, oat and almond plant‐based milks. Importantly, this included 47.3% of dairy consumers identifying a plant‐based milk as their primary milk of choice. Plant‐based milk popularity seen in this study are similar to those observed by IBISworld and Reeves who report soy and almond milk to be the most popular plant‐based milks in Australia, with oat milk recently increasing in popularity [47].

Our study identified trends in the motivations and perceptions behind plant‐based milk purchasing and international investigations have identified similar themes. Individuals who cared about health and the environment [6, 7, 8], and about animal welfare [7, 8], were more likely to choose plant‐based milk over cow's milk, as was found in our paper. A Danish study reported plant‐based milk consumers to be more likely than non‐plant‐based milk consumers to perceive that plant‐based milks are good for health, nutritionally equal or nutritionally better than cow's milk, and are environmentally friendly products [5], emulating the findings of our study. Furthermore, the participants demographics of these international studies reflected our own as they were primarily female, ranging from 59% to 81.8% of total participants within respective studies, and highly educated [5, 6, 7, 8]. This highlights the need for further investigation, both within Australia and internationally, to understand plant‐based milk motivations and perceptions from all sectors of the population.

Given the key role of dairy products providing essential nutrients in the Australian diet [19, 48], and a rapidly emerging trend for the replacement of cow's milk with plant‐based milk by consumers, it is timeous to investigate the nutritional adequacy of those choosing to follow a dairy free diet. Notably, non‐dairy consumers had a 15 g lower median intake of protein than those that consumed dairy, however, this did not reach statistical significance. Non‐dairy consumers within our study had significantly lower median iodine and vitamin B12 intakes than dairy consumers. Non‐dairy consumers also had increased fibre and lowered saturated fat intakes compared to dairy consumers, an intake trend that has previously been associated with vegetarian style eating patterns including those that exclude dairy products [49, 50]. Although increased fibre and decreased saturated fat consumption align more closely with dietary guidelines [51], decreased iodine and vitamin B12 intake may place consumers at increased risk of deficiency.

Previous Australian insight into the nutritional implications of plant‐based milk consumption in the context of the whole diet has been conducted through modelling techniques, and it is useful to examine if the findings of the current study reflect those of modelled investigations. Lawrence and colleagues examined the impact on nutrient intake if Australians were to substitute all cow's milk for plant‐based milk using dietary intake from the NNPAS of 2011–12 and using the AUSNUT food composition database [19]. The modelling resulted in Australians aged 2 years and older having decreased intakes of riboflavin, vitamin A, calcium, vitamin B12, iodine and phosphorus. However, as the present study is comprised of primarily female (79.3%), and younger individuals (median age 34 y Q1–Q3: 27–41 y), it is also useful to compare to the findings for women aged 19–35 years where modelling resulted in only vitamin B12 and iodine intake reductions of 10% and 15.4%, respectively [19]. The current study also saw this reduction in vitamin B12 and iodine in the reported intakes of participants. However, the current study observed no statistically significant decrease in any other micronutrient as seen in the modelling of the ‘all Australians 2 years and over’ category and did see a reduction in saturated fat and increase in fibre consumption that was not identified in any of the modelling by Lawrence and colleagues. Differences between the outcomes of the dietary modelling study and the current survey may be explained by the former only incorporating changes in milk type and not considering other dietary behaviour changes that may be closely associated with avoidance of dairy, such as omission of other animal products. Furthermore, the modelling by Lawrence and colleagues utilised data representative of the Australian population, whereas the current study demographics do not reflect that of the general Australian population [19].

One variable that may have influenced micronutrient intake of participants is the fortification of plant‐based milks consumed. When examined in our study, only 23.8% of 24‐h recall participants reported plant‐based milks were fortified with nutrients other than calcium, with 46.3% of participants providing insufficient information to determine fortification status. Insufficient information was mainly caused due to the number of products within the market, with any one brand providing multiple products within a plant‐based milk type that vary in fortification (e.g., almond, organic almond, barista almond, high protein almond, etc.) Due to this insufficient data, it is useful to discuss findings in comparison to the plant‐based milk products available within Australia. A 2023 audit into plant‐based milks available for purchase in Australia found that although 80.6% of plant‐based milks were fortified with at least calcium, only 27.1% and 3.1% of products were vitamin B12 and iodine fortified, respectively [18]. Although unable to accurately distinguish the fortification status of many of the plant‐based milks respondents consumed, it is reasonable to assume the majority were not iodine fortified, and many were not vitamin B12 fortified, suggesting this may have contributed to the decreased nutrient intake observed in this study.

Several observational studies in Australia and other high‐income countries identify trends in vitamin B12 and iodine intake for non‐dairy consumers that align with the findings of the current paper. A systematic literature review by Eveleigh and colleagues reported that vegans, compared to dairy‐consuming vegetarians and omnivores, tended to have the lowest iodine intake, along with the lowest urinary iodine concentration as an objective biomarker of iodine status [52]. Other systematic literature reviews have found that in general, all vegetarians, but particularly vegan individuals who do not supplement their diet with vitamin B12, have significantly lower intakes of vitamin B12 and are more likely to be vitamin B12 deficient when compared to individuals that follow omnivorous eating patterns [53, 54]. Our study identifies trends in iodine and vitamin B12 intake that are consistent with wider international literature. However, it is important to consider that our study did not collect adequate supplementation data and therefore, it is necessary to examine supplementation rates in this population. Previous research into the supplementation of Australian vegans and vegetarians shows that B12 is supplemented frequently at a rate of about 60%–70%, whereas iodine is far less readily supplemented in this group [55, 56]. Thus, non‐dairy consumers are at an increased risk of both vitamin B12 and iodine deficiency, however, iodine deficiency risk is compounded when considering supplementation rates.

Despite mandatory fortification of bread with iodised salt in Australia since 2009, women of childbearing age are still considered to be at increased risk of iodine deficiency [26]. In the current study of young women, we report similarly low iodine intakes, regardless of demographic or type of milk intake. However, investigations in larger sample sizes are required to confirm these findings. Recruitment of the sample may have resulted in participant bias, whereby, respondents may have been more likely to be women aged 18–50 and to follow vegetarian style eating patterns, two characteristics that are associated with lower iodine intakes [20, 21, 22, 23, 26, 52]. A similar trend has been reported using the UK Diet and Nutrition Survey (2014–17) data, whereby women of childbearing age who exclusively consumed plant‐based milk had similar median iodine intakes to cow's milk consumers [57]. Importantly, however, in that study, women of childbearing age who consumed plant‐based milks had a significantly lower UIC than cow's milk consumers [57].

There are several limitations to the current study. Nutritional supplement use was not considered in the 24‐h recall analysis and for some participants only one valid 24‐h recall was analysed, meaning that collected dietary data are less likely to accurately estimate dietary intake. A lower proportion of non‐dairy consumers (26%) completed two 24‐h recalls compared to 39% of dairy consumers which may have resulted in nutritional intake data being less representative of typical dietary intake in the former. As suggested by Subar and colleagues, we have focused on reporting the population means for nutrient intakes and using it as source of information on the types of plant‐based foods and beverages [58]. The targeted sampling methodology resulted in a predominantly, young, female, healthy weight and highly educated sample, which together with the small sample size that completed the dietary recall component of the study, limits generalisability of the findings. Furthermore, many of the non‐dairy consumers followed vegan or vegetarian style eating patterns that saw them limit other animal product‐containing food groups. Nevertheless, the findings are useful to identify trends of decreased vitamin B12 and iodine intake that can be examined in a more representative sample. The fortification status of plant‐based milks varied, and some participants did not provide sufficient information to determine fortification status. This variability could introduce inaccuracies in assessing the micronutrient intake of participants relying on fortified plant‐based milks. A strength of the study is the valuable information gained regarding the motivations of consumers who choose plant‐based milk by capturing a high proportion of non‐dairy consumers. This includes novel insight into Australian consumer motivations and perceptions behind plant‐based milk purchasing, being one of the first investigations into these consumer perceptions with transparent methodology.

To conclude, this study provides key insights into the consumption, motivations and perceptions of plant‐based milk in Australia, highlighting soy, almond and oat plant‐based milks as the most popular types. Key motivators for milk type choice were found to be animal rights, negative health symptoms not diagnosed by a health practitioner and environmental concerns. Further, non‐dairy consumers were more likely than dairy consumers to perceive plant‐based milks as healthier and better for the environment. When analysing the whole diet of dairy and non‐dairy consumers, average iodine, vitamin B12 and saturated fat intake were significantly lower, and fibre significantly higher, for non‐dairy consumers compared to dairy consumers. Non‐dairy consumers are at increased risk of vitamin B12 and iodine deficiency, two nutrients that are inconsistently fortified within Australian plant‐based milk. These findings underscore the importance of understanding consumer behaviour and dietary implications associated with milk choices in shaping public health and environmental policies, and it is recommended that fortification of plant‐based milk with iodine and vitamin B12 increases to support the health of those that choose to not consume dairy.

## Author Contributions

Isobel Harmer designed the survey, undertook data organisation, cleaning and analysis, drafted the original article and was responsible for the final manuscript. Joel C. Craddock, Anita Lawrence, Katherine Kent, Tracy McCaffery and Karen E. Charlton were involved in study conception, designing the study and interpretation of findings. Joel C. Craddock assisted with survey design and implementation, and review of vegan and vegetarian food substitutions. Katherine Kent contributed to data cleaning and analysis, and implementation of Intake24. Tracy McCaffery facilitated the use of the Intake24 software. All authors assisted in editing the paper, have critically reviewed its content and have approved the final version submitted for publication. This research was conducted by Isobel Harmer as part of a degree in Nutrition and Dietetics (Honours) at the University of Wollongong.

## Ethics Statement

Ethics approval was gained through the University of Wollongong Human Research Ethics Committee (HREC 2023/044).

## Conflicts of Interest

The authors declare no conflicts of interest.

## Supporting information

Supporting File

## Data Availability

The data that support the findings of this study are available from the corresponding author upon reasonable request.

## References

[1] Australian Bureau of Statistics , *Apparent Consumption of Selected Foodstuffs, Australia* (Australian Bureau of Statistics, 2022), https://www.abs.gov.au/statistics/health/health-conditions-and-risks/apparent-consumption-selected-foodstuffs-australia/2020-21.

[2] J. D. Flom and S. H. Sicherer , “Epidemiology of Cow's Milk Allergy,” Nutrients 11 (2019): 1051. 31083388 10.3390/nu11051051 PMC6566637

[3] T. M. Bayless , E. Brown , and D. M. Paige , “Lactase Non‐Persistence and Lactose Intolerance,” Current Gastroenterology Reports 19 (2017): 23. 28421381 10.1007/s11894-017-0558-9

[4] B. Yantcheva , S. Golley , D. Topping , and P. Mohr , “Food Avoidance in an Australian Adult Population Sample: The Case of Dairy Products,” Public Health Nutrition 19 (2016): 1616–1623. 26585823 10.1017/S1368980015003250 PMC10271172

[5] E. Martínez‐Padilla , I. Faber , I. L. Petersen , and E. Vargas‐Bello‐Pérez , “Perceptions Toward Plant‐Based Milk Alternatives Among Young Adult Consumers and Non‐Consumers in Denmark: An Exploratory Study,” Foods 12 (2023): 385. 36673476 10.3390/foods12020385 PMC9858389

[6] W. Su , Y. Y. Zhang , S. Li , and J. Sheng , “Consumers' Preferences and Attitudes Towards Plant‐Based Milk,” Foods 13 (2023): 2. 38201030 10.3390/foods13010002 PMC10778246

[7] K. S. McCarthy , M. Parker , A. Ameerally , S. L. Drake , and M. A. Drake , “Drivers of Choice for Fluid Milk Versus Plant‐Based Alternatives: What Are Consumer Perceptions of Fluid Milk?,” Journal of Dairy Science 100 (2017): 6125–6138. 28551193 10.3168/jds.2016-12519

[8] A. Boaitey and K. Minegishi , “Determinants of Household Choice of Dairy and Plant‐Based Milk Alternatives: Evidence From a Field Survey,” Journal of Food Products Marketing 26 (2020): 639–653.

[9] S. Chalupa‐Krebzdak , C. J. Long , and B. M. Bohrer , “Nutrient Density and Nutritional Value of Milk and Plant‐Based Milk Alternatives,” International Dairy Journal 87 (2018): 84–92.

[10] D. Escobar‐Sáez , L. Montero‐Jiménez , P. García‐Herrera , and M. C. Sánchez‐Mata , “Plant‐Based Drinks for Vegetarian or Vegan Toddlers: Nutritional Evaluation of Commercial Products, and Review of Health Benefits and Potential Concerns,” Food Research International 160 (2022): 111646. 36076378 10.1016/j.foodres.2022.111646

[11] K. E. Scholz‐Ahrens , F. Ahrens , and C. A. Barth , “Nutritional and Health Attributes of Milk and Milk Imitations,” European Journal of Nutrition 59 (2020): 19–34. 30937581 10.1007/s00394-019-01936-3

[12] S. K. Vanga and V. Raghavan , “How Well Do Plant Based Alternatives Fare Nutritionally Compared to Cow's Milk?,” Journal of Food Science and Technology 55 (2018): 10–20. 29358791 10.1007/s13197-017-2915-y PMC5756203

[13] D. Angelino , A. Rosi , G. Vici , et al., “Nutritional Quality of Plant‐Based Drinks Sold in Italy: The Food Labelling of Italian Products (FLIP) Study,” Foods 9 (2017): 682. 10.3390/foods9050682 PMC7278734 32466295

[14] K. A. Kopf‐Bolanz and A. Sousa , “Nutritional Implications of an Increasing Consumption of Non‐Dairy Plant‐Based Beverages Instead of Cow's Milk in Switzerland,” Journal of Advances in Dairy Research 5 (2017): 4.

[15] B. Walther , D. Guggisberg , R. Badertscher , et al., “Comparison of Nutritional Composition Between Plant‐Based Drinks and Cow's Milk,” Frontiers in Nutrition 9 (2022): 988707. 36386959 10.3389/fnut.2022.988707 PMC9650290

[16] N. W. Smith , A. C. Dave , J. P. Hill , et al., “Nutritional Assessment of Plant‐Based Beverages in Comparison to Bovine Milk,” Frontiers in Nutrition 9 (2022): 957486. 36003838 10.3389/fnut.2022.957486 PMC9394682

[17] Y. Y. Zhang , J. Hughes , and S. Grafenauer , “Got Mylk? The Emerging Role of Australian Plant‐Based Milk Alternatives as a Cow's Milk Substitute,” Nutrients 12 (2020): 1254. 32354190 10.3390/nu12051254 PMC7281999

[18] I. Harmer , J. C. Craddock , and K. E. Charlton , “How Do Plant‐Based Milks Compare to Cow's Milk Nutritionally? An Audit of the Plant‐Based Milk Products Available in Australia,” Nutrition & Dietetics 82 (2025): 76–85. 39344056 10.1111/1747-0080.12906 PMC11795225

[19] A. S. Lawrence , H. Huang , B. J. Johnson , and T. P. Wycherley , “Impact of a Switch to Plant‐Based Foods That Visually and Functionally Mimic Animal‐Source Meat and Dairy Milk for the Australian Population—A Dietary Modelling Study,” Nutrients 15 (2023): 1825. 37111044 10.3390/nu15081825 PMC10147004

[20] K. E. Charlton , L. Gemming , H. Yeatman , and G. Ma , “Suboptimal Iodine Status of Australian Pregnant Women Reflects Poor Knowledge and Practices Related to Iodine Nutrition,” Nutrition 26 (2010): 963–968. 20080029 10.1016/j.nut.2009.08.016

[21] K. Guttikonda , J. R. Burgess , K. Hynes , S. Boyages , K. Byth , and V. Parameswaran , “Recurrent Iodine Deficiency in Tasmania, Australia: A Salutary Lesson in Sustainable Iodine Prophylaxis and Its Monitoring,” Journal of Clinical Endocrinology & Metabolism 87 (2002): 2809–2815. 12050255 10.1210/jcem.87.6.8600

[22] J. E. Gunton , G. Hams , M. Fiegert , and A. McElduff , “Iodine Deficiency in Ambulatory Participants at a Sydney Teaching Hospital: Is Australia Truly Iodine Replete?,” Medical Journal of Australia 171 (1999): 467–470. 10615339 10.5694/j.1326-5377.1999.tb123749.x

[23] M. A. Hamrosi , E. M. Wallace , and M. D. Riley , “Iodine Status in Pregnant Women Living in Melbourne Differs by Ethnic Group,” Asia Pacific Journal of Clinical Nutrition 14 (2005): 27–31. 15734705

[24] Food Standards Australia and New Zealand , ed., *Standard 2.1.1 ‐ Cereal and Cereal Products* (Food Standards Australia and New Zealand, 2015).

[25] Food Standards Australia and New Zealand , *Proposal P1003 – Mandatory Iodine Fortification for Australia* (Food Standards Australia and New Zealand, 2008), https://www.foodstandards.gov.au/code/proposals/pages/proposalp1003mandato3882.aspx.

[26] K. Charlton , Y. Probst , and G. Kiene , “Dietary Iodine Intake of the Australian Population After Introduction of a Mandatory Iodine Fortification Programme,” Nutrients 8 (2016): 701. 27827915 10.3390/nu8110701 PMC5133088

[27] Australian Bureau of Statistics , *Microdata and TableBuilder: Australian Health Survey: Nutrition and Physical Activity* (Australian Bureau of Statistics, 2013), https://www.abs.gov.au/statistics/microdata-tablebuilder/available-microdata-tablebuilder/australian-health-survey-nutrition-and-physical-activity#cite-window1.

[28] S. Parkinson and L. Bromfield , “Recruiting Young Adults to Child Maltreatment Research Through Facebook: A Feasibility Study,” Child Abuse & Neglect 37 (2013): 716–720. 23768931 10.1016/j.chiabu.2013.04.009

[29] M. S. B. Yusoff , “ABC of Content Validation and Content Validity Index Calculation,” Education in Medicine Journal 11 (2019): 49–54.

[30] G. L. Trakman , A. Forsyth , R. Hoye , and R. Belski , “Developing and Validating a Nutrition Knowledge Questionnaire: Key Methods and Considerations,” Public Health Nutrition 20 (2017): 2670–2679. 28735598 10.1017/S1368980017001471 PMC10261290

[31] D. F. Polit , C. T. Beck , and S. V. Owen , “Is the CVI an Acceptable Indicator of Content Validity? Appraisal and Recommendations,” Research in Nursing & Health 30 (2007): 459–467. 17654487 10.1002/nur.20199

[32] Vabderbilt University , ed., *REDCap, 11.0.3* (Vabderbilt University, 2021).

[33] G. Eysenbach , “Improving the Quality of Web Surveys: The Checklist for Reporting Results of Internet E‐Surveys (CHERRIES),” Journal of Medical Internet Research 6 (2004): e34. 15471760 10.2196/jmir.6.3.e34 PMC1550605

[34] E. Simpson , J. Bradley , I. Poliakov , et al., “Iterative Development of an Online Dietary Recall Tool: INTAKE24,” Nutrients 9 (2017): 118. 28208763 10.3390/nu9020118 PMC5331549

[35] M. K. Rowland , A. J. Adamson , I. Poliakov , et al., “Field Testing of the Use of Intake24—An Online 24‐Hour Dietary Recall System,” Nutrients 10 (2018): 1690. 30404170 10.3390/nu10111690 PMC6266941

[36] C. Whitton , M. E. Rollo , C. E. Collins , et al., “Intake24 in Australia: Evaluating the Criterion Validity of a Self‐Administered 24‐Hour Recall Using a Controlled Feeding Study in Western Australia Adults,” Proceedings of the Nutrition Society 82 (2023): E84.

[37] P. Olivier and T. A. McCaffrey , “*Use of Intake24 in the Department of Health Intergenerational Health and Mental Health Study (IHMHS)* (Monash University, 2021).

[38] T. A. McCaffrey , E. Foster , and H. Ng , et al., *Intake 24‐Aus Food List* (Monash University, 2025).

[39] E. Foster , C. Lee , F. Imamura , et al., “Validity and Reliability of an Online Self‐Report 24‐Hour Dietary Recall Method (Intake24): A Doubly‐Labelled Water Study and Repeated Measures Analysis — CORRIGENDUM,” Journal of Nutritional Science 8 (2019): e41. 32042408 10.1017/jns.2019.38 PMC6984004

[40] C. Whitton , C. E. Collins , B. A. Mullan , et al., “Accuracy of Energy and Nutrient Intake Estimation Versus Observed Intake Using 4 Technology‐Assisted Dietary Assessment Methods: A Randomized Crossover Feeding Study,” American Journal of Clinical Nutrition 120 (2024): 196–210. 38710447 10.1016/j.ajcnut.2024.04.030 PMC11347807

[41] J. Bradley , E. Simpson , I. Poliakov , et al., “Comparison of INTAKE24 (an Online 24‐h Dietary Recall Tool) With Interviewer‐Led 24‐h Recall in 11–24 Year‐Old,” Nutrients 8 (2016): 358. 27294952 10.3390/nu8060358 PMC4924199

[42] D. A. Schoeller and M. S Westerterp‐Plantenga , *Advances in the Assessment of Dietary Intake* (CRC Press, 2017).

[43] Food Standards Australia and New Zealand , *AUSNUT 2011–13 Food Nutrient Database* (Food Standards Australia and New Zealand, 2011–2013).

[44] Food Standards Australia and New Zealand , *AUSNUT 2011–13 Food Recipe File* (Food Standards Australia and New Zealand, 2014).

[45] L. E. Marchese , G. A. Hendrie , S. A. McNaughton , P. G. Brooker , K. M. Dickinson , and K. M. Livingstone , “Comparison of the Nutritional Composition of Supermarket Plant‐Based Meat and Dairy Alternatives With the Australian Food Composition Database,” Journal of Food Composition and Analysis 129 (2024): 106017.

[46] J. C. Banna , M. A. McCrory , M. K. Fialkowski , and C. Boushey , “Examining Plausibility of Self‐Reported Energy Intake Data: Considerations for Method Selection,” Frontiers in Nutrition 4 (2017): 45. 28993807 10.3389/fnut.2017.00045 PMC5622407

[47] M. Reeves and IBISWorld, *Soy and Almond Milk Production in Australia* (IBISWorld, 2023).

[48] National Health and Medical Research Council , *Australian Dietary Guidelines – Summary* (National Health and Medical Research Council, 2013).

[49] N. Neufingerl and A. Eilander , “Nutrient Intake and Status in Adults Consuming Plant‐Based Diets Compared to Meat‐Eaters: A Systematic Review,” Nutrients 14 (2021): 29. 35010904 10.3390/nu14010029 PMC8746448

[50] G. K. Davey , E. A. Spencer , P. N. Appleby , N. E. Allen , K. H. Knox , and T. J. Key , “EPIC–Oxford: Lifestyle Characteristics and Nutrient Intakes in a Cohort of 33 883 Meat‐Eaters and 31 546 Non Meat‐Eaters in the UK,” Public Health Nutrition 6 (2003): 259–268. 12740075 10.1079/PHN2002430

[51] National Health and Medical Research Council , *Nutrient Reference Values for Australia and New Zealand* (National Health and Medical Research Council, 2018).

[52] E. R. Eveleigh , L. J. Coneyworth , A. Avery , and S. J. M. Welham , “Vegans, Vegetarians, and Omnivores: How Does Dietary Choice Influence Iodine Intake? A Systematic Review,” Nutrients 12 (2020): 1606. 32486114 10.3390/nu12061606 PMC7352501

[53] R. Pawlak , S. E. Lester , and T. Babatunde , “The Prevalence of Cobalamin Deficiency Among Vegetarians Assessed by Serum Vitamin B12: A Review of Literature,” European Journal of Clinical Nutrition 68 (2014): 541–548. 24667752 10.1038/ejcn.2014.46

[54] D. R. Bakaloudi , A. Halloran , H. L. Rippin , et al., “Intake and Adequacy of the Vegan Diet. A Systematic Review of the Evidence,” Clinical Nutrition 40 (2021): 3503–3521. 33341313 10.1016/j.clnu.2020.11.035

[55] J. C. Craddock , E. P. Neale , G. E. Peoples , and Y. C. Probst , “Examining Dietary Behaviours, Diet Quality, Motives and Supplementation Use in Physically Active Individuals Following Vegetarian‐Based Eating Patterns,” Nutrition Bulletin 47 (2022): 473–487. 36352440 10.1111/nbu.12592 PMC10098725

[56] A. J. Benham , D. Gallegos , K. L. Hanna , and M. T. Hannan‐Jones , “Vitamin B12 Supplementation Adequacy in Australian Vegan Study Participants,” Nutrients 14 (2022): 4781. 36432466 10.3390/nu14224781 PMC9695216

[57] M. Dineva , M. P. Rayman , and S. C. Bath , “Iodine Status of Consumers of Milk‐Alternative Drinks v. Cows' Milk: Data From the UK National Diet and Nutrition Survey,” British Journal of Nutrition 126 (2021): 28–36. 32993817 10.1017/S0007114520003876

[58] A. F. Subar , L. S. Freedman , J. A. Tooze , et al., “Addressing Current Criticism Regarding the Value of Self‐Report Dietary Data,” Journal of Nutrition 145 (2015): 2639–2645. 26468491 10.3945/jn.115.219634 PMC4656907

