# Supplementary material for: Understanding the Motivations, Perceptions and Nutritional Implications of Plant‐Based Milk Consumption Compared to Dairy‐Based Milk
Source: J Hum Nutr Diet. 2026 Apr 27;39:e70254. doi: 10.1111/jhn.70254 (PMC13121926; doi:10.1111/jhn.70254)
Supplement: Supplementary file 1 — Supporting File [file JHN-39-0-s001.docx]

**Supplementary Material**

**Supplementary 1: Written version of online survey utilised in study two.**

Please enter your email address *[short response text box]*

What is your gender?

What is your age?

What is your current postal code?

What is your Race/Ethnicity?

What is your height in centimetres (cm)?

What is your body weight in kilograms (kg)?

What is the highest level of education you have completed or enrolled in?

- Primary school
- I completed/am enrolled in high school
- I completed/am enrolled in a Diploma or equivalent
- University (I completed/am enrolled in a bachelor/undergraduate degree)
- University (I completed/am enrolled in a honours/master’s degree)
- University (I completed/am enrolled in a doctoral degree)
- Prefer not to say

Please select all the foods from the below list that you do NOT eat. If you eat all these foods, select “I eat all of the above” *[participants can select unlimited choices]*

- Red meat
- Poultry (chicken, turkey, duck, etc.)
- Fish/seafood
- Dairy products (cow’s milk, yoghurt, cheese)
- Eggs
- Honey
- I eat all of the above

Which of the following best aligns with your attitudes, values and beliefs around healthy eating?

- "I'm passionate about healthy eating & health plays a big part in my life. I use social media to follow active lifestyle personalities or get new recipes/exercise ideas. I may even buy superfoods or follow a particular type of diet. I like to think I am super healthy."
- "I'm health-conscious and being healthy and eating healthy is important to me. Although health means different things to different people, I make conscious lifestyle decisions about eating based on what I believe healthy means. I look for new recipes and healthy eating information on social media"
- "I aspire to be healthy (but struggle sometimes). Healthy eating is hard work! I've tried to improve my diet, but always find things that make it difficult to stick with the changes. Sometimes I notice recipe ideas or healthy eating hacks, and if it seems easy enough, I'll give it a go."
- "I try and live a balanced lifestyle, and I think that all foods are okay in moderation. I shouldn't have to feel guilty about eating a piece of cake now and again. I get all sorts of inspiration from social media like finding out about new restaurants, fun recipes and sometimes healthy eating tips."
- "I'm contemplating healthy eating but it's not a priority for me right now. I know the basics about what it means to be healthy, but it doesn't seem relevant to me right now. I have taken a few steps to be healthier but I am not motivated to make it a high priority because I have too many other things going on in my life."
- "I'm not bothered about healthy eating. I don't really see the point and I don't think about it. I don't really notice healthy eating tips or recipes and I don't care what I eat."
- Prefer not to say

Which of the following milks do you consume? (select all that apply)

- Full fat Cow's Milk
- Low fat Cow's Milk
- Skim milk Cow's milk
- Lactose free Cow's milk
- Almond milk
- Cashew milk
- Coconut milk
- Hemp milk
- Macadamia milk
- Oat milk
- Pea milk
- Rice milk
- Soy milk
- A combination milk (e.g. almond and coconut blend)
- I don't consume milk
- Other (please specify)

Which of the following milks do you most often consume? (select one)

- Full fat Cow's Milk
- Low fat Cow's Milk
- Skim milk Cow's milk
- Lactose free Cow's milk
- Almond milk
- Cashew milk
- Coconut milk
- Hemp milk
- Macadamia milk
- Oat milk
- Pea milk
- Rice milk
- Soy milk
- A combination milk (e.g. almond and coconut blend)
- I don't consume milk
- Other (please specify)

Do you know the brand of you chosen milk type that you are most likely to choose?

- Yes
- No
- I make my own milk

Which of the following are reasons that you drink the kind of milk/s that you do? (Select all that apply)

- I have always had the same type of milk
- Health
- Animal rights
- Weight loss
- Price
- Environmental concerns
- My religion or spiritual beliefs
- To improve my athletic performance
- Due to diagnosed intolerance/allergy/negative symptoms (e.g., bloating, diarrhoea, pain)
- Due to intolerance/allergy/negative symptoms (e.g., bloating, diarrhoea, pain) that have NOT been diagnosed
- My friends and/ or family do and it's easy for me
- My friends and/or family do so I feel pressure to as well
- It's trendy
- other
- prefer not to say

Which is the main reason that you drink the kind of milk/s that you do? Select the one option that you feel best represents your main priority reason

- I have always had the same type of milk
- Health
- Animal rights
- Weight loss
- Price
- Environmental concerns
- My religion or spiritual beliefs
- To improve my athletic performance
- Due to diagnosed intolerance/allergy/negative symptoms (e.g., bloating, diarrhoea, pain)
- Due to intolerance/allergy/negative symptoms (e.g., bloating, diarrhoea, pain) that have NOT been diagnosed
- My friends and/ or family do and it's easy for me
- My friends and/or family do so I feel pressure to as well
- It's trendy
- other
- prefer not to say

Is there any extra information you would like to share about why you choose to drink the milk that you do? leave blank if you have nothing else you would like to add. *[short response text box]*

For the following questions; plant milk refers to milks such as soy or almond etc. that have not come from an animal. Dairy milk refers to milk that has come from an animal, e.g., regular full fat cow's milk.

Do you think that plant milks are healthier than dairy milks?

- Yes, plant milks are healthier
- No, they are equally healthy
- No, dairy milk is healthier
- Other
- I don't know
- Prefer not to say

What do you think the health and nutrition differences, if any, are between plant milks and dairy milks? *[short response text box]*

Do you think that plant milks are better for the environment than dairy milk?

- Yes, plant milks are better
- No, they have equal impact
- No, dairy milk is better
- Other
- I don't know
- Prefer not to say

What information do you know about the environmental impact of plant milks and dairy milk? *[short response text box]*

Please check the 'yes' box below if you would like to be entered into our prize draw.
You will be automatically entered into the draw after you have completed both intake24 surveys. Complete the first one now, and a link to a second intake24 will be emailed to you in 8 days time.

Winners will be informed via email once the survey closes (within 3 months).

- Yes
- No

Click "Link to Intake24" below to complete the first (of two) Intake 24 questionnaire, which asks you to report the type and quantities of all food and drink that you have consumed in the previous day (from when you woke up until your last meal/snack/beverage).

If you provide details about the plant-based milk you consume, please mention the specific brand name of the milk you drank when you search for it. For example, instead of simply typing "oat milk," please enter the brand name "Oatley." This will help the researchers match the accurate nutritional information to the milk you consumed. While searching, you might come across various options, including the generic name like "oat milk," which is acceptable to choose. However, the brand name you initially searched for will be saved automatically.

Following submission of the first Intake24 questionnaire, you will be sent out an invitation to complete the second intake24 by email 8 days later. This will once again ask you to consider all food and drinks consumed in the previous day.

Thank you for participating in this research.

Please feel free to share the survey with your own networks and share with anyone you think who may be interested learning more!

**Supplementary 2: Survey long response analysis**

Table A: Key themes that emerged when respondents asked “what do you think the health and nutrition differences, if any, are between plant milks and dairy milk?”.

| Theme | Exemplifying Quotation |
| --- | --- |
| Differing nutrient composition | *“plant based milks can be matched to be as healthy as cows milk (aka fortification) thought many don't even try to do this making them not as healthy or good dairy alternative”* – 38yo female dairy consumer |
| Negative health attributes of dairy milk | *“Cow's milk is made to turn a 30kg calf into 100kg by weaning... that's not what an adult human should be drinking” –*37yo female that does not consume dairy |
| Lactose intolerance | *“Plant based milks are better as a whole due to the lower calorie count, lactose free (about 70% of the population can't digest it)…”* – 23yo female that does not consume dairy |
| Concern of highly processed nature of some plant based milks | *“Plant based milk although dairy free have other nasty ingredients like vegetable oil, canola oil that are awful for the body.”* – 37yo female that does not consume dairy |

*Differing nutrient composition*

Many participants identified that a dominant nutrition difference between plant based milk and dairy relate to variations in nutrient composition. The most mentioned of these was the improved fat content of plant based milk. Respondents expressed knowledge that plant based milk has a lower fat content and less cholesterol and saturated fat than CM.

“*Less saturated fat and cholesterol in plant based milks*.” – 36-year-old male that does not consume dairy products.

Some participants also voiced awareness that plant based milks contain lesser amounts of energy and protein and many respondents were aware of lower micronutrient content.

“*I believe typically plant milks are lower kJ, protein and micronutrients*” – 24-year-old female dairy product consumer.

Many participants across both dairy and non-dairy consumers were able to identify that the amount of calcium that naturally exists in plant based milks is lower than CM. Other nutrients identified to be in lower amounts in plant based milk mentioned by some respondents, generally dairy consumers, were iodine, vitamin B12 and vitamin D. Further, it was commonly mentioned across both dairy and non-dairy consumers that plant based milk can be fortified to provide the nutrients that cow’s milk contains. This was almost always looked at from a positive viewpoint, as something that is adding value to plant based milk from a health perspective.

*“Milk from animals has iodine, vitamin d, calcium, etc. but fortified plant milks can be just as good and have other health benefits”* – 46-year-old female that does not consume dairy

*“plant based milks can be matched to be as healthy as cows milk (aka fortification) though many don't even try to do this making them not as healthy or good dairy alternative”* – 38-year-old female dairy product consumer

Several respondents, across both dairy and non-dairy consumers, were also aware that there are nutritional differences between plant based milk types, other than fortification status. In particular, many identified that soy milk contained more protein that other plant based milks.

“*Each different type of milk would have nutritional differences.*” – 29-year-old female that does not consume dairy

*“Apart from Soy milk, most plant milks are low in protein.”* – 30-year-old male that does not consume dairy

*Negative Health Attributes of dairy*

Some non-dairy consumers, and a handful of dairy consumers, voiced considerable concern over animal bi-products that they believe are present in CMs. These include hormones that may interact with human endocrine systems, and cow pus, blood and faeces.

“*No added mammalian oestrogens in plant milks. Plus no accidental faeces, or blood, or pus included…”*- 34-year-old female that does not consume dairy products.

Another common theme amongst some non-dairy consumers is a very strong concern over negative long term health outcomes that are associated with dairy consumption. These include bone degradation and increased osteoporosis risk as well as increased cancer risk.

*“… Cows milk has been heavily linked to osteoporosis, greater risks of bone fractures ect...”* – 23-year-old female that does not consume dairy

Many non-dairy consumers also believe that CM is not designed for human consumption and therefore, is not appropriate.

*“Cow's milk is made to turn a 30kg calf into 100kg by weaning... that's not what an adult human should be drinking” –*37-year-old female that does not consume dairy

*Lactose intolerance*

Non-dairy consuming respondents commonly mentioned the lack of lactose in plant based milks as a positive health attribute, often without specifically mentioning lactose intolerance. Of these respondents, only some included reasons for zero lactose being positive, with common reasons being that lactose is difficult for humans to process and can cause negative symptoms such as bloating.

*“Plant based milks are better as a whole due to the lower calorie count, lactose free (about 70% of the population can't digest it)…”* – 23-year-old female that does not consume dairy

*Concern of highly processed nature of some plant based milks*

A sub-group of both dairy and non-dairy consuming participants indicated they perceive highly processed plant based milks and those that contain extra additives such as oils and emulsifiers as not as healthy.

*“Plant based milk although dairy free have other nasty ingredients like vegetable oil, canola oil that are awful for the body.”* – 37-year-old female that does not consume dairy

*“…many plant milks are Ultra-processed. it's pretty hard to find minimally processed plant-based milks”* – 28-year-old female that consumes dairy products

Table B: Key themes that emerged when participants asked: “what information do you know about the environmental impact of plant milks and dairy milk?”.

| Theme | Exemplifying Quotation |
| --- | --- |
| Resource use | *“Far fewer resources (water, land) required to produce any plant milk, compared to dairy.”* – 51yo female that does not consume dairy |
| Carbon emissions | *“The carbon and water footprints of plant based milks is much smaller than those of animal milks.”* – 44yo female that does not consume dairy |
| Destruction of physical environment | *“Dairy farms pollute soil and waterways”* – 41yo female that does not consume dairy |
| Elements considered equal between milk types | *“Both dairy and non-dairy milk have the impact of packaging and transport”* – 76yo female that does not consume dairy |
| Almond milk | *“Plant milks generally better except almond”* – 29yo female dairy product consumer |

Overall, respondents almost always perceived that plant based milks have a reduced impact on the environment in comparison to CM production. Further, there appeared no obvious difference in common themes from dairy and non-dairy consuming participants.

*Resource use*

Many participants report that dairy production requires more water, land and resources in general to support cattle. Respondents also noted that cows require feed, such as soybeans, that requires additional land to grow, or that could be used to directly feed humans.

*“Far fewer resources (water, land) required to produce any plant milk, compared to dairy.”* – 51-year-old female that does not consume dairy

*Carbon Emissions*

Several participants identified that dairy produces more GHG emission than plant based milk, with some noting methane production as a specific negative contributor to climate change.

*“The carbon and water footprints of plant based milks is much smaller than those of animal milks.”* – 44-year-old female that does not consume dairy

*Destruction of physical environment*

Some respondents indicated that CM causes more destruction of the physical environment including the pollution of ecosystems and waterways and deforestation for crop growth or grazing land.

*“Dairy farms pollute soil and waterways”* – 41-year-old female that does not consume dairy

*“Need more cows/animals for more milk, causing more deforestation for said cattle farms + crop farms specifically to feed the cattle”* – 22-year-old female that does not consume dairy

*Elements considered equal between milk types*

Some respondents identified elements of both plant based milk and CM production that are equally detrimental to the environment. These include the processing and manufacturing required to produce the beverages, packaging of the beverages and the transportation of goods.

*“All require processing so will have some sort of environmental impact either way”* – 27-year-old female dairy product consumer

*“Both dairy and non-dailry milk have the impact of packaging and transport”* – 76-year-old female that does not consume dairy

*Almond milk*

Many respondents consider almond milk the worst plant based milk for the environment, even sometimes being viewed as worse than CM. The most commonly reported reason for this is increased water requirements.

*“Plant milks generally better except almond”* – 29-year-old female dairy product consumer

*“…almond milk is the worst in terms of the impact on water”* – 28-year-old non-binary gender identifying dairy product consumer
